# Sample size and number of outcome measures of veterinary randomised controlled trials of pharmaceutical interventions funded by different sources, a cross-sectional study

**DOI:** 10.1186/s12917-017-1207-0

**Published:** 2017-10-04

**Authors:** K. J. Wareham, R. M. Hyde, D. Grindlay, M. L. Brennan, R. S. Dean

**Affiliations:** 1 Centre for Evidence-based Veterinary Medicine, School of Veterinary Medicine and Science, The University of Nottingham, Sutton Bonington campus, Loughborough, LE12 5RD UK; 2 Centre of Evidence Based Dermatology, The University of Nottingham, King’s Meadow Campus Lenton Lane, Nottingham, NG7 2NR UK

**Keywords:** Quality, Primary outcome, Study design and data analysis, Clinical trials, Evidence based medicine

## Abstract

**Background:**

Randomised controlled trials (RCTs) are a key component of the veterinary evidence base. Sample sizes and defined outcome measures are crucial components of RCTs.

To describe the sample size and number of outcome measures of veterinary RCTs either funded by the pharmaceutical industry or not, published in 2011.

**Methods:**

A structured search of PubMed identified RCTs examining the efficacy of pharmaceutical interventions. Number of outcome measures, number of animals enrolled per trial, whether a primary outcome was identified, and the presence of a sample size calculation were extracted from the RCTs. The source of funding was identified for each trial and groups compared on the above parameters.

**Results:**

Literature searches returned 972 papers; 86 papers comprising 126 individual trials were analysed. The median number of outcomes per trial was 5.0; there were no significant differences across funding groups (*p* = 0.133). The median number of animals enrolled per trial was 30.0; this was similar across funding groups (*p* = 0.302). A primary outcome was identified in 40.5% of trials and was significantly more likely to be stated in trials funded by a pharmaceutical company. A very low percentage of trials reported a sample size calculation (14.3%).

**Conclusions:**

Failure to report primary outcomes, justify sample sizes and the reporting of multiple outcome measures was a common feature in all of the clinical trials examined in this study. It is possible some of these factors may be affected by the source of funding of the studies, but the influence of funding needs to be explored with a larger number of trials. Some veterinary RCTs provide a weak evidence base and targeted strategies are required to improve the quality of veterinary RCTs to ensure there is reliable evidence on which to base clinical decisions.

**Electronic supplementary material:**

The online version of this article (10.1186/s12917-017-1207-0) contains supplementary material, which is available to authorized users.

## Background

Randomised controlled trials (RCTs) are one of the main sources of evidence that can be directly used for clinical decision-making around treatment choice. They do however vary in quality, both in conduct and reporting, a problem which can reduce their reliability and usefulness to decision-makers. One important feature of trials is which, and how many, outcomes (or endpoints) are used to assess the effectiveness of an intervention. An outcome can be defined as:

*“A component of a participant's clinical and functional status after an intervention has been applied, that is used to assess the effectiveness of an intervention.”* (www.cochrane.org/glossary)

All outcomes should be relevant and specific to the question of interest being addressed, and, in almost all cases, should be pre-specified prior to the trial starting and reported in the trial protocol. Any post hoc outcome measures should have a clear rationale for their inclusion. Ideally, one single primary outcome (the outcome considered to be the most important to relevant stakeholders, and which the study is powered for) should be identified, with other outcomes of interest being clearly identified as secondary [1, 2]. Previous assessments of the veterinary clinical trials literature, based on a number of sampling strategies for trial selection, have demonstrated varied reporting standards of outcome measures and identification of primary outcomes. The proportion of trials that identify a primary outcome in previous studies of veterinary RCTs vary from 2% to 83% [3–7]. Understanding the current state of, and potential deficiencies in, outcome reporting is crucial to improve trial design, execution, and reporting and therefore increase the reliability of clinical trial results. The factors that affect the quality of outcome reporting need to be identified if improvements are to be made.

Another crucial factor in the reliability of RCT results is whether the study was adequately powered to detect a difference between two or more variables of interest within the study. Reporting guidelines for RCTs advocate reporting of a sample size calculation to determine the number of subjects required to detect a clinically relevant difference in a specified (primary) outcome between two trial groups [1, 2]. Sample size calculations have been shown in the past to be vastly underreported in the veterinary trials literature [3–7].

In a recent study, we reported that the source of funding (pharmaceutical company or other) has an effect on positive outcome reporting (sponsorship bias) in veterinary clinical trials [8]. The impact of funding on the size of trials and whether a sample size calculation has been undertaken remains unclear in medical RCTs [9–11]. To date, there has been no report of the influence of funding source on the size of veterinary RCTs and whether primary outcomes are stated or a sample size calculation has been undertaken. This study was done concurrently with Wareham et al. [8] to examine further whether funding source has an effect on other aspects of trial design and delivery.

The aim of this study is to describe the number of outcome measures reported, the animals included, whether primary outcome measures are stated and sample size calculations are performed in a sample of veterinary pharmaceutical single dose efficacy RCTs either funded by the pharmaceutical industry or not, published in a single calendar year (2011). The effect of funding source on these aspects of trial design and delivery was then explored.

## Methods

A cross-sectional study of RCTs was conducted. The target population was feline, canine, equine, bovine and ovine RCTs where a pharmaceutical agent was the intervention of interest and efficacy was assessed. The sample population was feline, canine, equine, bovine and ovine RCTs published in 2011 within journals indexed in PubMed. This study was conducted in parallel with Wareham et al. [8] and the search strategy and literature filtering processes reported in full in that manuscript also apply to this study The same sample of literature was used for data extraction in both studies [8].

### Search strategy and filtering of results

A structured search of PubMed was conducted in June 2013 using the “clinical trial” Publication Type combined with the relevant species MeSH heading e.g. “clinical trial” [publication type] AND cats [mh]. This was done for each of the 5 species studied: cats, dogs, horses, cattle and sheep. The search was limited to one calendar year with a PubMed filter: 01/01/11 – 31/12/11. Search results were exported into EndNote® software for filtering. Papers indexed as RCTs by PubMed (“randomised controlled trials” [publication type]) were extracted and confirmed if, when reviewed by the authors, they were RCTs according to the Cochrane definition below (http://www.cochrane.org/glossary/):

**“**An experiment in which two or more interventions, possibly including a control intervention or no intervention, are compared by being randomly allocated to participants. In most trials one intervention is assigned to each individual but sometimes assignment is to defined groups of individuals (for example, in a household) or interventions are assigned within individuals (for example, in different orders or to different parts of the body).”

All publications containing RCTs, published in 2011, and relevant to the species of interest were then categorised into four intervention subcategories based on the main intervention of interest of the study (Table 1 - Level 1 exclusion criteria):

1. Pharmaceutical – consisting of an active pharmaceutical ingredient
2. Nutritional
3. Para-pharmaceutical – including probiotics, prebiotics, synbiotics, nutraceuticals and supplements/vitamins/minerals if not considered part of the total dietary ration
4. Other – including surgical interventions, management/husbandry interventions, non-medicinal shampoos, studies relating to diagnostic tests.

**Table 1:** Two levels of inclusion and exclusion criteria applied to the search results

Level 1: Inclusion criteria for publications	Level 1: Exclusion criteria for publications
Species of interest is cats, dogs, horses, cattle or sheep	Not about cats, dogs, horses, cattle or sheep
Published in 2011	E published only in 2011 if full publication occurred in a different calendar year
RCT according to PubMed publication types and the Cochrane definition	Not an RCT (not indexed as an RCT by PubMed or not fulfilling Cochrane definition of an RCT)
Treatment of interest is a pharmaceutical intervention	Treatment of interest is not a pharmaceutical agent e.g. nutritional, surgical, animal husbandry etc
Level 2: Inclusion criteria for analysis of pharmaceutical RCTs	Level 2: Exclusion criteria for analysis of pharmaceutical RCTs
Primary aim is to assess efficacy	Primary aim was not to assess efficacy (pharmacokinetic/dynamic studies, safety studies, physiological effects, resistance testing, testing routes of administration only, testing timing of administration only)
Identifiable treatment or protocol of interest	Treatment or protocol of interest could not be identified
Single dose of the treatment of interest used	Multiple doses of the treatment of interest used/dose finding studies
Published in English	Not available in English

Only publications within the ‘Pharmaceutical intervention’ subcategory were included in this study; these were assessed for further eligibility for analysis according to the second level of inclusion and exclusion criteria in Table 1.

Publications included in the analysis were therefore single dose efficacy studies of pharmaceutical interventions in cats, dogs, horses, cattle or sheep published in 2011. The single dose trials were selected for analysis as it was then possible to be certain an outcome was assigned to a single treatment. The trials included animals with naturally occurring and experimentally induced disease. A single calendar year was chosen to ensure it was feasible to complete this work within the timeframe of the funding in a situation where the total number of veterinary RCTs published per year is unknown. The year 2011 was chosen, as it was hoped that by the time the searching was done (June 2013) the 2011 trials would be fully indexed therefore possible to locate in PubMed and any erratum reported. In the case of a publication containing more than one trial, each trial was included independently in the analysis if it met all inclusion criteria.

### Sources of funding

For each included trial the source of funding was categorised as one of the following:

1. Pharmaceutical company funding stated or pharmaceutical company involvement (e.g. drug donated by a pharmaceutical company or authors associated with a pharmaceutical company) (P)
2. Non-pharmaceutical company funding stated (NP)
3. No funding source stated (NF)

### Outcome recording

A data extraction form (Additional file 1) was used by the authors. Outcomes mentioned in the materials and methods section of the manuscripts were included in the analysis; outcomes that were reported as results but not mentioned in the methods were not included. Outcomes that had multiple components (e.g. complete blood count, serum biochemistry, and meat yield and meat quality grade assessments) were classed as a single outcome each unless specific features were relevant to the disease, in which case these were extracted as individual outcomes.

The following information was extracted:

1. **Total number of outcome results:** Every result for each unique outcome measure is included in this total. Where multiple treatment and control groups were used, each group containing the treatment of interest (either alone or in combination) was compared to its relevant control group for each outcome measure i.e. each unique outcome measure could actually be recorded more than once within this total. For example, a trial measuring heart rate and respiratory rate, which compared two different treatment groups to a control group, would have a total number of 4 outcome results.
2. **Number of unique outcome measures:** The number of individual outcome measures mentioned in the methods section of the manuscripts, i.e. each mentioned outcome measure included only once in this total. For example, a trial measuring heart rate and respiratory rate would have a unique number of 2 outcome measures.
3. **Primary outcome measure:** If the primary outcome measure was stated this was recorded. Trials reporting only one outcome measure, or completing a sample size calculation for one specific outcome measure were automatically categorised as having identified a primary outcome.
4. **Number of animals:** The number of animals enrolled in each trial was recorded.
5. **Sample size calculation:** Reporting of a sample size calculation was recorded as ‘yes’ or ‘no’. Post-hoc power calculations were recorded as ‘no’.

All assessments made throughout the study were agreed upon by two authors (KW and RH/RD) with any disputes resolved by a third author (RD/RH). The authors who undertook the assessments of the studies are all veterinary surgeons with methodological expertise in study design and critical appraisal.

### Statistical analysis

Categorical data were presented descriptively as raw numbers and percentages. Numerical discrete data that were not normally distributed were presented as median, inter-quartile range (IQR) and range (R). Within a data set, if some subsets of data were non-normally distributed, all were presented as median, IQR and R. Statistical comparisons between funding categories for the number of outcomes measured, number of unique outcome measures, and the number of animals in the trials, were made using an independent samples Kruskal-Wallis test with a significance level of 0.05. Statistical comparisons between funding categories for whether a primary outcome was stated or not and whether a sample size calculation was done or not were made using Fishers Exact test with a significance level of 0.05. The NP and NF groups were sometimes combined and compared to the P group (the group od interest) as there were far more trials stated to be funded by pharmaceutical companies than other sources, Results for different species are described only and were not compared statistically due to small group sizes. All statistical analyses were conducted in IBM SPSS Version 21.

## Results

### Study numbers

Initial searches returned 972 papers across the five species studied. Following application of the two levels of exclusion criteria (Table 1), 86 papers reporting 126 individual trials remained for data extraction and analysis (Table 2, Additional file 2; for full details see 20). Of the 126 trials, 86 (68%) were in the pharmaceutical funding category, 19 (15%) were in the non-pharmaceutical funding category and the remaining 21 (17%) were in the ‘no funding source stated’ category.

**Table 2:** Number and funding source of papers and individual trials following level 2 exclusion criteria application

	Number of cat papers (trials)	Number of dog papers (trials)	Number of horse papers (trials)	Number of cattle papers (trials)	Number of sheep papers (trials)	Total number of papers (trials, % of total trials)
Papers including pharmaceutical agent RCTs	17	49	28	61	17	172
Papers excluded from analysis	9	21	17	29	10	86
Papers analysed	8 (9 trials)	28 (44 trials)	11 (11 trials)	32 (36 trials)	7 (26 trials)	86 (126 trials)
Funding sources of analysed papers						
Pharmaceutical company funded/pharmaceutical company involvement	4 (5 trials)	17 (33 trials)	4 (4 trials)	20 (23 trials)	2 (21 trials)	47 (86 trials; 68%)
Non pharmaceutical funding stated	3 (3 trials)	4 (4 trials)	2 (2 trials)	6 (7 trials)	3 (3 trials)	18 (19 trials; 15%)
No funding stated	1 (1 trial)	7 (7 trials)	5 (5 trials)	6 (6 trials)	2 (2 trials)	21 (21 trials; 17%)

Included studies are the pharmaceutical agent RCTs. Table reproduced from REF companion manuscript

### Number of outcomes reported

#### Total number of outcome results

The median total number of outcome results reported per trial was 5.0, with a large overall range (IQR = 3.0–11.0, Range = 1.0–36.0, *N* = 126, Table 3). For individual funding categories, the highest number of outcomes reported per trial (*N* = 9.0) occurred in the non-pharmaceutical funding category (IQR = 3.0–16.0, Range = 2.0–24.0) compared to 5.0 (IQR = 3.0–9.3, Range = 1.0-36.0) in the pharmaceutical category and 6.0 (IQR = 2.5–14.5, Range = 1.0–26.0) in the ‘no funding source declared’ category. The differences between funding categories were not significant (*p* = 0.133).

**Table 3:** Number of outcomes reported per trial for funding categories and individual species

		Median	Interquartile range	Range
Species	Cats (*N* = 9)	10.0	5.5–14.0	2.0–24.0
	Dogs (*N* = 44)	4.5	2.0–5.0	1.0–36.0
	Horses (*N* = 11)	12.0	7.0–14.0	1.0–26.0
	Cattle (*N* = 36)	5.5	3.0–11.8	1.0–36.0
	Sheep (*N* = 26)	5.0	3.0–8.5	1.0–18.0
Funding category	Trials with pharmaceutical funding/involvement (*N* = 86)	5.0	3.0–9.3	1.0–36.0
	Trials with non-pharmaceutical funding stated (*N* = 19)	9.0	3.0–16.0	2.0–24.0
	Trials with no funding source stated (*N* = 21)	6.0	2.5–14.5	1.0–26.0
All funding categories and species combined	All trials (*N* = 126)	5.0	3.0–11.0	1.0–36.0

#### Number of unique outcome measures

Overall, the median number of unique outcome measures per trial (discounting instances where the same outcome measure was recorded more than once) were lower but largely similar to the results for the total number of outcomes reported (Median = 5.0, IQR = 3.0–8.0, Range = 1.0–36.0, Additional file 2: Table S1). The differences between funding categories were not significant (*p* = 0.26).

### Primary outcome identification

Overall, 51 out of the 126 trials (40.5%) defined a primary outcome (Table 4). A significantly larger proportion of all trials within the pharmaceutical funding category identified a primary outcome compared to the other funding groups (*P* = 42/86, 48.8%; NP = 4/19, 21.1%; NF = 5/21, 23.8%; *p* = 0.01). The proportions of trials defining a primary outcome did vary from this pattern across the different species, with sheep having the largest proportion of trials identifying a primary outcome, and horses the lowest (Table 4).

**Table 4:** Number and percentages of trials identifying a primary outcome for individual species and funding categories

	Trials with pharmaceutical funding/involvement	Trials with non-pharmaceutical funding stated	Trials with no funding source stated	All trials
Cats	2/5 (40%)	1/3 (33.3%)	1/1 (100%)	4/9 (44.4%)
Dogs	8/33 (24.2%)	2/4 (50%)	1/7 (14.3%)	11/44 (25%)
Horses	2/4 (50%)	0/2 (0%)	0/5 (0%)	2/11 (18.2%)
Cattle	11/23 (47.8%)	1/7 (14.3%)	2/6 (33.3%)	14/36 (38.9%)
Sheep	19/21 (90.5%)	0/3 (0%)	1/2 (50%)	20/26 (76.9%)
All species	42/86 (48.8%)	4/19 (21.1%)	5/21 (23.8%)	51/126 (40.5%)

### Number of animals per trial

Overall the median number of animals enrolled per trial was 30, with a large associated range (IQR = 17.5–98.8, Range = 5–5996, Table 5). The median number of animals enrolled per trial was broadly similar and not significantly different between all the funding categories (*P* = 30.0, NP = 24.0, NF = 42.0, *p* = 0.302; Table 5). The number of animals enrolled per trial was similar across all the species examined except for cattle where it was noticeably higher (cats = 32.0, dogs = 27.5, horses = 26.0, cattle = 233.0 and sheep = 20.0; Table 5).

**Table 5:** Numbers of animals enrolled per trial for individual species and funding categories

		Median	Interquartile range	Range
Species	Cats (*N* = 9)	32.0	21.5–93.0	16.0–121.0
	Dogs (*N* = 44)	27.5	16.0–43.0	5.0–316.0
	Horses (*N* = 11)	26.0	15.0 – 61.0	6.0–373.0
	Cattle (*N* = 36)	233.0	35.0–943.0	8.0–5996.0
	Sheep (*N* = 26)	20.0	19.0–24.8	10.0–655.0
Funding category	Trials with pharmaceutical funding/involvement (*N* = 86)	30.0	19.8–125.8	7.0–5996.0
	Trials with non-pharmaceutical funding stated (*N* = 19)	24.0	14.0–69.0	8.0–369.0
	Trials with no funding source stated (*N* = 21)	42.0	16.0–75.0	5.0–1031.0
All funding categories and species combined	All trials (*N* = 126)	30.0	17.5–98.8	5.0–5996.0

### Sample size calculations

Within the 126 trials, only 18 (14.3%) reported performing a sample size calculation. The proportions of trials across the funding groups reporting a sample size calculation were low and very similar, with no statistical difference across the funding groups (PH = 12/86, 14.0%; NP = 3/19, 15.8%; NF = 3/21, 14.3%, *p* = 0.9). Again, there were differences across the species in the proportions within each category ranging from 0% of sheep trials, to 27.8% of cattle trials reporting a sample size calculation (Table 6).

**Table 6:** Numbers and percentages of trials reporting sample size calculations for individual species and funding categories

	Trials with pharmaceutical funding/involvement	Trials with non-pharmaceutical funding stated	Trials with no funding source stated	All trials
Cats	1/5 (20%)	0/3 (0%)	1/1 (100%)	2/9 (22.2%)
Dogs	2/33 (6.1%)	2/4 (50%)	1/7 (14.3%)	5/44 (11.4%)
Horses	0/4 (0%)	0/2 (0%)	1/5 (20%)	1/11 (9.1%)
Cattle	9/23 (39.1%)	1/7 (14.3%)	0/6 (0%)	10/36 (27.8%)
Sheep	0/21 (0%)	0/3 (0%)	0/2 (0%)	0/26 (0%)
All	12/86 (14.0%)	3/19 (15.8%)	3/21 (14.3%)	18/126 (14.3%)

## Discussion

This sample of 126 veterinary RCTs reported multiple outcomes (median of 5 per trial) assessed on a relatively small number of (median 30) animals per trial. Less than half of the trials specified a primary outcome in their materials and methods and sample size calculations were rarely performed. Of these measures only whether a primary outcome measure was defined or not was significantly affected by the source of funding.

The influence that the source of funding has on the reporting of RCTs was recently reported in a related manuscript [8] and it was found that trials with pharmaceutical company involvement were more likely to have positive outcomes of treatment (sponsorship bias) Wareham et al. found that across all trials, regardless of funding, it was found that due to poor reporting and/or conduct of the trials an unclear risk of bias was highly prevalent [8]. In the current study only whether a primary outcome was reported or not seemed to be significantly affected by funding source. However the current study is a descriptive hypothesis generating study and the number of trials in some of the funding categories may be subject to both Type I and Type II errors. The reasons behind why funding source may affect how outcomes are measured and reported and the size of trials are unclear. In medical RCTs, there has been some suggestion that funding source may influence the size of trials across a number of different pharmaceutical, surgical and nursing interventions, so further investigation in the veterinary field may be warranted [9–11].

A median value of 5 outcomes per trial is lower than has been previously reported in similar studies of veterinary clinical trials. Other reports include a mean of 10.8 outcomes per trial in a study examining dog and cat clinical trials [5], and a median of 8.5 outcomes per trial in livestock clinical trials [3]. These studies were published in 2010 and 2009 respectively and a comparison of their results with the ones in this study could be interpreted as a general reduction in the number of outcomes reported per trial over time. Alternatively, methodological differences between the previous studies and this one could explain the findings. For example, the studies all include differing subsets of literature (e.g. clinical trials versus RCTs, any intervention versus pharmaceutical interventions only, different ranges of species included) and employ different data extraction methods. Interestingly, the non-pharmaceutical funding group in the current study had a similar median number of outcomes per trial (9.0) to these previous studies, with the pharmaceutical funding group having a lower median (5.0), although the ranges are wide and differences not significant within this sample. The maximum number of outcomes reported per trial was 36. One concern with having large numbers of outcomes in a trial is that statistically, the ability to accurately detect a difference between two groups diminishes as an increasing number of statistical tests are performed on a limited amount of data (and number of subjects), that is, the chance of introducing ‘error’ into the results increases [12]. However, if a trial is well designed and adequately powered a primary outcome and a number of secondary outcomes can be investigated if the sample size, statistical analysis are appropriate. In this study, trials involving cats and horses appeared to have a higher median number of outcomes per trial compared to the other species; however the small sample sizes make drawing conclusions from this problematic.

Selecting and properly defining a primary outcome (in advance of conducting a trial) is extremely important, and is outlined in the CONSORT RCT reporting guideline and the adapted livestock version REFLECT [1, 13, 14]. In the current study, 40% of trials specified a primary outcome and trials in the pharmaceutical funding category more frequently identified a primary outcome compared to the other groups. Previous studies have reported extremely varied findings on this topic with the number of trials defining a primary outcome varying from 86% of small animal RCTs [4] to 7% in dog and cat clinical trials [5]. If primary and secondary outcomes are not pre-specified and adhered to, there is the risk that throughout a trial, the most ‘interesting’ results can be reported rather than those the trial was designed to address. On the other hand, outcomes that prove to be ‘negative’ in respect to the treatment of interest may not be reported, biasing the evidence base. Such ‘outcome switching’ is currently being tracked in human trials literature by the COMPARE initiative [15]. Without a trials registry, where protocols can be made available before a trial begins, it is extremely difficult to track and address this issue.

Primary outcomes are also important as they are the measures used to determine the number of animals required for the trial, via a sample size calculation. The low percentage of authors reporting that they have undertaken a sample size calculation in this study is actually higher than previously found; reports have varied from 0 to 18% [3–7] which again could reflect a genuine improvement over time, or a difference in the sample of literature studied. The number of animals actually enrolled per trial encompassed a very large range (5-5996) with noticeably higher numbers of animals enrolled in cattle trials compared to the other species potentially reflecting the easier recruitment of large numbers of animals within herds to a trial. When a sample size calculation is not reported, the reader cannot easily identify if the number of subjects in the trial was sufficient to detect a difference between two intervention groups, even if such a difference exists [16], or if the identification of a difference between two groups is reliable. Conversely, excessively large sample sizes are an ethical concern as more participants are exposed to potential risks than is necessary [2, 16]. Whilst an adequately powered study is the ideal situation, there are advocates of the theory that some evidence is better than no evidence, and smaller trials can be performed and later combined in evidence syntheses to contribute substantially to the evidence base. There are certain caveats to this approach, which are that trials must be of sound methodological design to eliminate bias, and it is extremely important that all trials are published so they are available for evidence synthesis [17].

The proportions of trials defining a primary outcome, and particularly those reporting a sample size calculation, were vastly lower in the current study, and others in the veterinary literature, compared to those in the medical literature [18]. There are still huge improvements to be made; one way of improving how well veterinary RCTs are reported is through the endorsement and increased awareness of relevant reporting guidelines (e.g. CONSORT, REFLECT) by both authors and journals. Another approach to improve trial conduct, increase the transparency of RCTs, and reduce publication bias, is through compulsory registration of trial protocols, as advocated by the ‘All-Trials’ and ‘Veterinary All-Trials’ campaigns (www.alltrials.net), [19]. A veterinary clinical trials unit comprising a large network of practices would also be of huge benefit to the veterinary profession. It would enable access to large numbers of patients, records and provide centralised expertise and advice allowing the generation of high quality, relevant evidence. A cross-discipline approach where academia and industry work more closely together could also be beneficial for raising the overall standards of veterinary trials.

### Strengths and limitations

To the authors knowledge, in veterinary medicine no-one has yet investigated the role of funding on the reporting of outcomes and size of RCTs involving veterinary species. This unique manuscript compliments Wareham et al. [12] and describes the potential role of funding in study design. The manuscript also provides further insight into outcome reporting and size of trials on a different sample of veterinary RCTs compared to those already published [3–7].

One major limitation of this study is the imbalance in trial numbers across the funding categories due to the large proportion of trials in the pharmaceutical funding group, resulting in the number of trials in the non-pharmaceutical funding group and the ‘no funding source declared’ group being relatively small. Of particular concern is that studies encompassing more than one trial (for example, one sheep study comprised 19 individual trials) tended to be in the pharmaceutical funding category and could have caused clustering of the data. Another limitation is that authors were not blinded to manuscript details while extracting data. Time and financial constraints limited this study to one calendar year of publications, which did not yield enough trials to rule exclude the possibility of Type II errors hence this is primarily a descriptive report of these RCTs. Hence a larger study, powered using the data from this study incorporating more years of publications would be beneficial; in particular this could allow potential species specific differences to be explored. In addition, only Medline was searched and it has been shown previously that Medline does not include all clinical veterinary literature [20] however, it does have functionality that enables RCTs to be identified. This does mean that not all trials in 2011 that met our inclusion criteria would have been found. An inherent problem for all studies investigating the quality of veterinary RCTs is the reliance on good reporting to enable the investigators to adequately analyse the trials.

## Conclusions

The results of this study are important to those who use the results of pharmaceutical trials to inform practice and to researchers undertaking RCTs with veterinary species. The findings contribute to the body of knowledge indicating that veterinary clinical trials could be substantially improved in terms of reporting, and almost certainly design and execution, irrespective of funding source. It is difficult to accurately assess the true strength of trial conduct when they are poorly reported. Ultimately, the low quantity and quality of evidence available to veterinary practitioners severely inhibits our ability to practice in an evidence-based way; improvements are essential to ensure the veterinary profession has a sound scientific knowledge base on which to make clinical decisions, ensuring the best care for patients.

## Additional files

Additional file 1: Data extraction tool 310,117 BLANK. (XLSX 13 kb)

Additional file 2: Table S1. Number of unique outcome measures reported per trial for funding categories and individual species. References for all papers included in the analysis within this study. (DOCX 152 kb)

## Abbreviations

CONSORT: Consolidated Standards for Reporting Trials
IQR: Interquartile range
NF: Group of trials for which no funding source was stated
NP: Group of trials for which non-pharmaceutical funding was stated
P: Group of trials for which pharmaceutical funding/involvement was stated
R: Range
RCT: Randomised controlled trial
REFLECT: Reporting guidelines for randomised control trials for livestock and food safety
