# Supplementary material for: Sample size and number of outcome measures of veterinary randomised controlled trials of pharmaceutical interventions funded by different sources, a cross-sectional study
Source: BMC Vet Res. 2017 Oct 4;13:295. doi: 10.1186/s12917-017-1207-0 (PMC5628436; doi:10.1186/s12917-017-1207-0)
Supplement: Supplementary file 2 — Number of unique outcome measures reported per trial for funding categories and individual species. References for all papers included in the analysis within this study. (DOCX 152 kb) [file 12917_2017_1207_MOESM2_ESM.docx]

**TITLE**

Sponsorship bias and quality of randomised controlled trials in veterinary medicine

Wareham KJ, Hyde RM, Grindlay D, Brennan ML and Dean RS.

Centre for Evidence-based Veterinary Medicine, School of Veterinary Medicine and Science, The University of Nottingham, Sutton Bonington campus, Loughborough, LE12 5RD, UK

Kathryn.wareham@nottingham.ac.uk

Rachel.dean@nottingham.ac.uk

Marnie.brennan@nottingham.ac.uk

Douglas.grindlay@nottingham.ac.uk

Robert Hyde: svxrh1@exmail.nottingham.ac.uk

**Corresponding author:** R Dean, Centre for Evidence-based Veterinary Medicine, School of Veterinary Medicine and Science, University Of Nottingham, Sutton Bonington campus, Loughborough, LE12 5RD, rachel.dean@nottingham.ac.uk

**ABSTRACT**

**Background:** Randomised controlled trials (RCTs) are considered the gold standard form of evidence for assessing treatment efficacy, but many factors can influence their reliability including methodological quality, reporting quality and funding source.

The aim of this study was to examine the relationship between funding source and positive outcome reporting in veterinary RCTs published in 2011 and to assess the risk of bias in the RCTs identified.

**Methods**: A structured search of PubMed was used to identify feline, canine, equine, bovine and ovine clinical trials examining the efficacy of pharmaceutical interventions published in 2011. Funding source and outcomes were extracted from each RCT and an assessment of risk of bias made using the Cochrane risk of bias tool.

**Results:** Literature searches returned 972 papers, with 86 papers (comprising 126 individual RCTs) included in the analysis. There was found to be a significantly higher proportion of positive outcomes reported in the pharmaceutical funding group (P) compared to the non-pharmaceutical (NP) and ‘no funding source stated’ (NF) groups (P = 56.9%, NP = 34.9%, NF = 29.1%, p<0.05). A high proportion of trials had an unclear risk of bias across the five criteria examined.

**Conclusions:** We found evidence that veterinary RCTs were more likely to report positive outcomes if they have pharmaceutical industry funding or involvement. Consistently poor reporting of trials, including non-identification of funding source, was found which hinders the use of the available evidence.

**Keywords**

Clinical trials, study design and data analysis, evidence based medicine, risk of bias

**Background**

In order to effectively practice veterinary medicine in an evidence-based way, it is imperative that accurate scientific evidence is available so that the evidence base is complete, reliable, and therefore not misleading. Randomised controlled trials (RCTs), along with their synthesis in the form of systematic reviews, are considered to be the gold standard method for assessing the efficacy of treatment interventions and are a valuable source of information on which to base clinical decisions [[1](#_ENREF_1)]. The results of RCTs can however be affected by many biases including selection, performance, detection, attrition and reporting biases [[2](#_ENREF_2), [3](#_ENREF_3)]. The presence of bias can lead to misinterpretation of treatment efficacy or harms, and mislead clinicians when putting the evidence into practice.

Sponsorship bias (the influence of funding source on the reporting of trial results) is an additional potential problem when assessing the reliability of RCTs. The medical literature contains differing reports over whether financial conflicts of interest influence the reported results of a trial. Some studies report a greater likelihood of positive results for industry funded trials [[4](#_ENREF_4), [5](#_ENREF_5)], while some report no difference between industry and non-industry sponsored trials [[6](#_ENREF_6), [7](#_ENREF_7)]. A recent overview of medical literature in a Cochrane systematic review concluded that drug and medical device studies were more likely to report favourable results when the study was sponsored by a manufacturer [[8](#_ENREF_8)].

There have been several studies examining the methodological and reporting quality of clinical trials in the published veterinary literature [[9-11](#_ENREF_9)]. Such studies have highlighted issues with the reporting of RCTs and have shown how these reporting deficiencies are associated with an increased likelihood of a trial reporting one or more positive outcomes [[10](#_ENREF_10)]. To our knowledge, no studies to date have examined the influence of funding source on the likelihood of reporting positive outcomes in the veterinary RCT literature.

The aim of this study was to examine the relationship between funding source and proportions of positive outcome reporting in veterinary RCTs involving a pharmaceutical intervention published in a single calendar year (2011). A secondary aim was to assess the risk of bias of veterinary RCTs published in the same time period.

**Methods**

A cross-sectional study of veterinary RCTs was conducted. The target population was feline, canine, equine, bovine and ovine RCTs where a pharmaceutical agent was the intervention of interest and efficacy was assessed. The sample population was feline, canine, equine, bovine and ovine RCTs published in 2011 within journals indexed in PubMed.

***Search Strategy and Filtering of Results***

A structured search of PubMed was conducted in June 2013 using the “clinical trial” Publication Type combined with the relevant species MeSH heading e.g. “clinical trial” [publication type] AND cats [mh]. This was done for each of the 5 species studied: cats, dogs, horses, cattle and sheep (Figure 1). The search was limited to one calendar year with a PubMed filter: 01/01/11 – 31/12/11. Search results were exported into EndNote® software for filtering. Papers indexed as RCTs by PubMed (“randomised controlled trials” [publication type]) were extracted, investigators then confirmed if they were RCTs according to the Cochrane definition below (<http://www.cochrane.org/glossary/>):

**“**An experiment in which two or more interventions, possibly including a control intervention or no intervention, are compared by being randomly allocated to participants. In most trials one intervention is assigned to each individual but sometimes assignment is to defined groups of individuals (for example, in a household) or interventions are assigned within individuals (for example, in different orders or to different parts of the body).”

All publications containing trials confirmed by the investigators as being RCTs, published in 2011, and relevant to the species of interest were then categorised into four intervention subcategories based on the main intervention of interest of the study (Table 1 - Level 1 exclusion criteria):

1. Pharmaceutical – consisting of an active pharmaceutical ingredient, including anthelmintics and vaccines
2. Nutritional
3. Para-pharmaceutical – including probiotics, prebiotics, synbiotics, nutraceuticals and supplements/vitamins/minerals if not considered part of the total dietary ration
4. Other – including surgical interventions, management/husbandry interventions, non-medicinal shampoos, studies relating to diagnostic tests.

Only publications within the ‘Pharmaceutical intervention’ subcategory were included in this study; these were assessed for further eligibility for analysis according to the second level of inclusion and exclusion criteria in Table 1.

**TABLE 1 HERE (all tables at the end of the manuscript)**

Publications included in the analysis were therefore single dose efficacy studies of pharmaceutical interventions in cats, dogs, horses, cattle or sheep published in 2011. In the case of a publication containing more than one trial, each trial was included independently in the analysis if it met all inclusion criteria.

***Sources of funding***

For each included trial the source of funding was categorised as one of the following:

1. Pharmaceutical company funding stated or pharmaceutical company involvement (e.g. drug donated by a pharmaceutical company or authors associated with a pharmaceutical company) (P)
2. Non-pharmaceutical company funding stated (NP)
3. No funding source stated (NF)

***Outcome recording***

All outcomes mentioned in the methods section of the manuscripts were extracted and the result for each outcome was recorded. Outcomes that were reported as results but not mentioned in the methods were not included in the analysis. The result for each outcome was recorded in one of the five categories below (adapted from [[10](#_ENREF_10)]):

- 1. Treatment of interest had a statistically significant positive effect on the outcome
     - Treatment better than any control group
     - Treatment equal to positive control group (whether non-inferiority/equivalence design or not)
     - Safety/lack of adverse effects equal to, or better than, any control group
  2. Treatment of interest had a statistically significant negative effect on the outcome
     - Treatment worse than any control group
     - Treatment equal to negative control group
     - Safety/adverse effects worse than any control group
     - Treatment equal to a positive control group in a superiority analysis
  3. No significant difference between treatment and control groups
     - Outcome remained constant throughout the study (no measurable effect of treatment on the outcome)
  4. Results for the outcome were described only
     - There was data reported for an outcome that could have been statistically analysed, but no analysis was presented (if an outcome did not occur in any group, e.g. adverse events, it was treated as having been statistically analysed)
     - Outcomes such as descriptions of pathological appearances with no numerical data attached.
  5. Results for the outcome were not reported

Outcome measures that had multiple components (e.g. complete blood count and serum biochemistry, meat yield and meat quality grade assessments) were classed as a single outcome each unless specific features were relevant to the disease, in which case these were extracted as individual outcomes. If an outcome had a result recorded at multiple time points, an overall judgement was made as to which of the above categories was most appropriate (i.e. the outcome was only recorded once regardless of how many time points it was measured). Where multiple treatment and control groups were used, each group containing the treatment of interest (either alone or in combination) was compared to its relevant control group for each outcome.

***Risk of bias assessment***

All the included studies were assessed at the study level using the Cochrane risk of bias tool [[2](#_ENREF_2)]. The five features assessed were: random sequence generation, allocation concealment, blinding, incomplete outcome data and selective outcome reporting. Following the Cochrane guidelines for the risk of bias tool each category was assessed as being at a high, low or unclear risk of bias. These features allow the risks of selection bias, performance bias, detection bias, attrition bias and reporting bias to be assessed (see Additional file 1 for definitions of these types of bias). We did not include the category of ‘Other bias’ from the tool.

All assessments made throughout the study were agreed upon by two authors (KW and RH/RD) with any disputes resolved by a third author (RD/RH).

***Statistical analysis***

Categorical data were presented descriptively as raw numbers and percentages. Associations between funding source and positive outcome reporting were analysed using a Pearson’s chi squared test and Bonferroni post hoc test with adjusted p values. Significance level was set at p<0.05. Results for different species are described only and were not compared statistically due to small group sizes. All statistical analyses were conducted in IBM SPSS Version 21.

**Results**

***Overall study numbers***

A total of 972 papers were retrieved from the initial searches (96 for cats, 255 for dogs, 135 for horses, 371 for cattle and 115 for sheep; Figure 1). Following an initial review and exclusions based on year of publication in paper copy and species of interest there were 410 papers given the Publication Type for RCTs in PubMed; 390 of which were confirmed to be RCTs according to the Cochrane definition. Of these, 172 papers (172/390, 44.1%) were describing RCTs in which the treatment of interest was a pharmaceutical intervention and were included in further analysis (Figure 1). The remainder comprised nutritional studies (121/390, 31.0%), para-pharmaceutical agent studies (17/390, 4.4%) and ‘other RCTs’ (80/390, 20.5%).

Following application of the second set of exclusion criteria to the RCT pharmaceutical intervention studies, 86 papers remained in the study from which outcomes, bias and sources of funding were extracted (Figure 1, Table 2 and Additional Table 1). Eleven papers (all except one of which were within the pharmaceutical funding group) reported more than one RCT, notably one sheep paper reported 19 separate RCTs. As each trial was assessed individually as a separate entry, there were 126 trials included in the full analysis (Table 2 and Additional file 2 for full references of the publications analysed).

Of these 126 trials, 86 (68.3%) were funded by the pharmaceutical industry or had pharmaceutical company involvement, 19 trials (15.1%) explicitly stated they were not funded by the pharmaceutical industry, and 21 trials (16.7%) did not state any source of funding within the manuscript (Table 2).

**TABLE 2 HERE**

***Funding source and outcome reporting***

From the 126 trials included in the analysis, a total of 960 outcomes were extracted. Overall, 47.5% of outcomes (456/960) recorded in the trials were statistically positive compared to 28.8% (276/960) which were recorded as being statistically negative; 1.9% of outcomes (18/960) remained unchanged during the study (no significant difference category), 14.7% of outcomes (141/960) were described only and 7.2% (69/960) were not reported at all in the results (Table 3).

Between funding groups there were significant differences in the proportions of outcomes recorded in each of the outcome categories (Table 3, Pearsons chi squared, p<0.001). The proportion of positive outcomes reported was significantly higher in the pharmaceutical group than in the non-pharmaceutical and ‘no funding source stated’ groups (P = 56.9%, NP = 34.9%, NF = 29.1%, p<0.05). Correspondingly, there was a significantly lower proportion of negative outcomes recorded for the pharmaceutical group compared to the other two groups (P = 23.5%, NP = 37.6%, NF = 37.1%, p<0.05). Across all funding groups the proportion of outcomes recorded as ‘no significant difference’ was low, however the ‘no funding group’ had a significantly higher proportion compared to the pharmaceutical group (NF = 4.6%, P = 0.8%, p<0.05); the non-pharmaceutical group was not different to either of the other two groups (NP = 2.6%, p>0.05). There were no significant differences between the funding groups in the proportion of ‘described only’ or ‘not reported’ outcomes (p>0.05).

The above analysis categorised a treatment group which had equal results to a positive control group as a ‘positive’ outcome, even if the study did not use a non-inferiority design. If these results were instead considered to be in a ’no significant difference’ category, the pattern of significantly higher positive, and lower negative, outcome reporting in the pharmaceutical group compared to the other two groups was still present (p<0.05).

**TABLE 3 HERE**

***Risk of bias assessment***

Of the 126 included trials, the majority (92/126, 73.0%) were assessed as having an unclear risk of selection bias as there was inadequate or no description of how randomisation sequences were generated and employed. The vast majority of the trials were assessed as having an unclear risk of bias for allocation concealment (109/126, 86.5%) as it was impossible to determine what procedures had been followed. Blinding was reported more consistently, with 44 of the 126 trials (34.9%) being assessed as having a low risk of bias, 72/126 (57.1%) having an unclear risk, and the remaining 10 (7.9%) having a high risk of bias. Around half of the trials (65/126, 51.6%) were at low risk of bias for incomplete outcome reporting. There was a high risk of bias for incomplete outcome reporting in 19 out of the 126 trials (15.1%) due to missing data, or lack of analysis of the full population of animals randomised in the trial. Twenty-nine of the 126 trials (23.0%) were judged to be at a high risk of bias for selective outcome reporting, only 10/126 (7.9%) were at an unclear risk of bias, and the remaining 87 (69.0%) were assessed as being at a low risk of bias (Figure 2 and Table 4).

The results of comparing the quality criteria across the trials in different funding are shown in Table 4. The highest percentage of unclear risk for sequence generation was in the pharmaceutical group where 67 out of 86 trials (77.9%) were judged to be at an unclear risk of bias with a lower proportion in the non-pharmaceutical group (12/19, 63.2%) and 3/21 (61.9%) in the no funding declared group (13/21, 61.9%). The pharmaceutical group also had a higher proportion of unclear risk for incomplete outcome reporting in comparison to the other two funding groups (P = 36/86, 41.9%, NP = 3/19, 15.8%, NF = 3/21, 14.3%) and a correspondingly lower proportion of trials in the low risk category for this criteria. The high risk for selective outcome reporting was seen across all the funding categories (P=18/86, 20.9%; NP=5/19, 26.3%; NF= 6/21, 28.6%), however the pharmaceutical group had the largest proportion of studies in the low risk category for this criteria compared to the other groups (P = 64/86, 74.4%, NP = 11/19, 57.9%, NF = 12/21, 57.1%). Similar distributions of risk for blinding and allocation concealment were seen across the funding groups (Table 4).

**TABLE 4 HERE**

**Discussion**

This study found a significantly higher proportion of positive outcomes reported in RCTs with pharmaceutical funding (56.9%) or involvement compared to those with declared non-pharmaceutical funding (34.9%) or with no funding source stated (29.1%) within the sample of literature studied. There was a correspondingly lower proportion of negative outcomes reported in trials within the pharmaceutical funding group (23.5%) compared to the other two groups (37.6% and 37.1%). When assessing the trials for risk of bias across the five main categories using the Cochrane risk of bias tool, a large proportion were at an ‘unclear’ risk indicating significant reporting deficiencies. A high risk of bias was most predominantly seen for selective outcome reporting (reporting bias), and more moderately for incomplete outcome data (attrition bias) and blinding (detection bias). Proportions of trials at high, low or unclear risk of bias for the different quality criteria were largely similar across funding categories.

The sponsorship bias detected in this study is in accordance with many reports in the medical literature where an association between funding source and positive results has been demonstrated, most notably in a Cochrane Review of drug and medical devices [[8](#_ENREF_8)]. There are many reasons why such a bias may be present in the published literature including differences in the methodological quality of trials; inherent biases in trial conduct to favour a treatment; a genuinely greater likelihood that pharmaceutical companies would be testing pharmaceutical agents that are likely to perform well; and inadequacies in trial reporting which favour a treatment. Additionally, publication bias may play a role through researchers within different environments potentially being more or less likely to publish trials demonstrating a positive effect compared to trials showing a ‘negative’ result. Further studies are required to examine this finding and its potential causative factors in more detail, in particular whether there are correlations between quality criteria and funding source, something which this study did not investigate.

There are a variety of methods that could have been utilised for the current study. For example, in medical literature reviewing the presence of sponsorship bias, it is common to report one overall conclusion for a paper (i.e. overall the paper has a positive/negative/not significantly different outcome) determined either by the reviewers, based on the assertions of the authors or on the statistical analysis of one primary outcome of the study [[4](#_ENREF_4), [7](#_ENREF_7), [12](#_ENREF_12)]. The method we have used, whereby we have extracted each outcome and its result, is more achievable in the veterinary literature, as primary outcomes are often unspecified [[10](#_ENREF_10), [13](#_ENREF_13)], but different results would potentially be obtained using a different approach. Of note in this study is the potential for differences between species, and potential clustering of some types of trials, e.g. anthelmintic efficacy trials, to have skewed the data; these limitations will be discussed in more detail below. To date, we have found no other publications examining the association of funding source with positive outcome reporting in the veterinary literature with which to compare our results. The group of trials with no funding source stated are particularly difficult to assess in this study as no assumptions can be made as to which of the two other groups they would most appropriately belong to. Within the results, they appear to be most like the non-pharmaceutical group of trials in their characteristics, but this in itself highlights a continuing problem of poor reporting of clinical trials (20% of trials in this study did not report a funding source).

Selective outcome reporting, for example not reporting, or incompletely reporting, results for pre-specified outcomes, or reporting outcomes that were not pre-specified, can introduce reporting bias into a study and influence the overall results [[2](#_ENREF_2), [3](#_ENREF_3)]. A striking feature of our data was the high proportion of outcomes that were described only (18.9%) or were mentioned in the materials and methods then not reported in the results (10.3%). This could partly be due to manuscripts not detailing clearly which of the parameters being measured were intended to be outcomes used to assess efficacy, leading us to misclassify the information, highlighting again the issue of poor reporting. A previous study reporting quality criteria and outcome data from a sample of dog and cat trials also reported a high percentage of outcomes with no formal statistical analysis (31%) and a lower percentage not reported at all (3.1%) [[10](#_ENREF_10)]. The proportions of outcomes in these two categories contribute to the overall high risk of reporting bias (selective outcome reporting) found in this study. Research has shown that outcomes that are not reported, or incompletely reported are more likely to be statistically insignificant [[14](#_ENREF_14), [15](#_ENREF_15)]. This highlights the need for pre-specified primary and secondary outcomes to be explicitly stated in the methods and adhered to when reporting results. One approach which should help to combat this problem is for all clinical trial protocols to be registered in advance, so a comparison can be made with the final report; this approach is being championed by the AllTrials campaign in human medicine. AllTrials aims to ensure that all clinical trials are registered before they commence and that all are fully reported [[16](#_ENREF_16), [17](#_ENREF_17)]. A similar initiative is currently underway for veterinary clinical trials [[18](#_ENREF_18)]; these schemes should also help to combat publication bias. Publication bias, meaning negative studies are less likely to be published than positive ones, is a problem that has been identified across scientific publishing generally and which can lead to over estimates of treatment effects [[3](#_ENREF_3), [15](#_ENREF_15)]. The potential impact of publication bias on our study results would depend on who was funding any unpublished trials.

The high proportions of ‘unclear’ risk of bias for the five quality criteria assessed in this study indicate a significant issue with poor reporting, a feature which has also been described in previous quality assessments of veterinary clinical trial literature [[9](#_ENREF_9), [10](#_ENREF_10), [13](#_ENREF_13)]. This does not necessarily equate to poor methodological trial conduct, but a lack of complete reporting means that the methodology cannot be adequately assessed [[19](#_ENREF_19), [20](#_ENREF_20)]. This study did not set out to assess the impact of risk of bias on levels of positive outcome reporting. However, it has previously been shown in both veterinary and medical literature that incomplete or inadequate reporting of certain quality criteria (e.g. method of randomisation) is linked to an exaggeration of treatment efficacy [[10](#_ENREF_10), [13](#_ENREF_13), [21](#_ENREF_21), [22](#_ENREF_22)].

The CONSORT reporting guideline was developed in order to improve the reporting of RCTs, making it easier to ascertain what was done, identify possible sources of bias, and evaluate the reliability of a study [[23](#_ENREF_23), [24](#_ENREF_24)]. In general, the adoption of the CONSORT checklist has improved the reporting of RCTs in the medical literature, but there are still reporting deficits [[25](#_ENREF_25), [26](#_ENREF_26)]. In veterinary medicine the REFLECT statement is also available, which is an extension to the CONSORT reporting guideline specifically developed for RCTs involving livestock [[27](#_ENREF_27), [28](#_ENREF_28)]. Strict adherence to such reporting guidelines [[29](#_ENREF_29)] should have reduced all the ‘unclear’ assessments of bias made in this study and would have allowed us to identify the funding source of all the trials. Most importantly, this would allow more reliable assessments of treatment efficacies to be made, meaning more effective translation of evidence into clinical practice. A recent survey assessing the awareness of reporting guidelines amongst veterinary editors reported that 35.1% of journal editors said reporting guidelines were referred to in their instructions to authors [[30](#_ENREF_30)]. An improvement in the endorsement of reporting guidelines by journals could help to improve the reporting quality of the veterinary clinical trial literature as it has done for medicine.

A significant limitation of this study is that there were a relatively small number of trials included in the analysis, and due to the large proportion of pharmaceutical trials in the sample (68%), the groups for comparison were unbalanced and the non-pharmaceutical group small. Another, larger study would be extremely beneficial in assessing the presence of sponsorship bias in the veterinary clinical trials literature. In particular, an exploration of potential differences between species, or between companion animal versus production animals, warrants further investigation with larger sample sizes (no significant differences were found in the current study, see Table 2). Results of this type of study can be very dependent on the methods, including what types of studies are included (e.g. we have only included pharmaceutical interventions), which outcome classifications are used, the way in which outcomes are extracted (e.g. we did not include results for outcomes which were not mentioned in the materials and methods) and how funding categories are divided, meaning results across studies could be very different. Another limitation of this study is that the authors were not blinded to any manuscript details during data extraction potentially leading to biased interpretation. The lack of inclusion of efficacy studies where multiple doses of the test treatment were used is another significant limitation of the study. On balance it was felt that inclusion of these could potentially skew the results due to multiple entries for the trial by including each dose, or selecting only one of the doses. The inclusion of multiple trials within one publication may also skew results, as the methods, and therefore assessment of quality, tend to be identical for all the included trials. As most multiple trial papers were in the pharmaceutical category, this could potentially lead to clustering of information. Of particular influence in this study were RCTs assessing anthelmintic agents as these often contained multiple similar trials with an overwhelming proportion of positive outcomes. As they fulfilled our initial inclusion criteria they remained in our sample but their impact on the overall results may be substantial. The subjective assignment of a single outcome result for an outcome which was assessed at multiple time points is another limitation which was necessary for practicality. Limits to the initial sample size were needed due to cost and time constraints; a single calendar year search in PubMed was chosen to give a representative, recent sample of trials, rather than selecting certain journals to search. Using PubMed also allowed us to search by publication type. Not including studies unavailable in English was a necessary cost and time limitation but only one paper was excluded on this basis so this is unlikely to have affected the study outcomes.

**Conclusions**

This study found a positive association between pharmaceutical funding or involvement and increased positive outcome reporting. Consistently poor reporting of trials, including non-identification of funding source was identified, which hinders the assessment and use of the limited evidence available to the profession.

**Abbreviations**

RCT – randomised controlled trial

CONSORT – Consolidated Standards for Reporting Trials

P – group of trials for which pharmaceutical funding/involvement was stated

NP – group of trials for which non-pharmaceutical funding was stated

NF – group of trials for which no funding source was stated

**Declarations**

***Ethics approval and consent to participate***

This research was approved by the Ethics Committee of the School of Veterinary Medicine and Science at the University of Nottingham.

***Consent for publication***

Not applicable

***Availability of data and materials***

The datasets analysed during the current study are available from the corresponding author on reasonable request.

***Competing interests***

The Centre for Evidence-based Veterinary Medicine (CEVM) is supported by an unrestrictive grant from Elanco Animal Health and The University of Nottingham. Three of the authors (KW, DG and RD) were funded by this grant, MB by the University of Nottingham and RH was an undergraduate veterinary student at the University Of Nottingham and then worked in private practice during the completion of this work. KW is currently employed on a research grant from Elanco Animal Health.

***Funding***

This work was supported by an unrestrictive grant from Elanco Animal Health and The University of Nottingham. The topic of study, study design, statistical analysis, interpretation of the results, decision to publish and writing of the manuscript were undertaken independently of all funders of the CEVM.

***Authors’ contributions***

All authors were involved in the design of the research project. DG, KW and RH designed the searching strategies. KW, RH and RD extracted and analysed the data. All authors were involved in interpreting the analysed data. KW wrote the draft manuscript. All authors contributed to editing the manuscript. All authors read and approved the final manuscript.

***Acknowledgements***

Not applicable

**References**

1. Balshem H, Helfand M, Schünemann HJ, Oxman AD, Kunz R, Brozek J, Vist GE, Falck-Ytter Y, Meerpohl J, Norris S, Guyatt GH: GRADE guidelines: 3. Rating the quality of evidence. Journal of Clinical Epidemiology 2011; 64(4):401-406.

2. Higgins JP, Altman DG, Gotzsche PC, Juni P, Moher D, Oxman AD, Savovic J, Schulz KF, Weeks L, Sterne JA: The Cochrane Collaboration's tool for assessing risk of bias in randomised trials. BMJ (Clinical research ed) 2011; 343:d5928.

3. Gluud LL: Bias in Clinical Intervention Research. American Journal of Epidemiology 2006; 163(6):493-501.

4. Leopold SS, Warme WJ, Fritz Braunlich E, Shott S: Association between funding source and study outcome in orthopaedic research. Clinical orthopaedics and related research 2003(415):293-301.

5. Flacco ME, Manzoli L, Boccia S, Capasso L, Aleksovska K, Rosso A, Scaioli G, De Vito C, Siliquini R, Villari P, Ioannidis JP: Head-to-head randomized trials are mostly industry sponsored and almost always favor the industry sponsor. J Clin Epidemiol 2015; 68(7):811-820.

6. Naci H, Dias S, Ades AE: Industry sponsorship bias in research findings: a network meta-analysis of LDL cholesterol reduction in randomised trials of statins. BMJ : British Medical Journal 2014; 349.

7. Pang WK, Yeter KC, Torralba KD, Spencer HJ, Khan NA: Financial conflicts of interest and their association with outcome and quality of fibromyalgia drug therapy randomized controlled trials. Int J Rheum Dis 2015; 27(10):12607.

8. Lundh A, Sismondo S, Lexchin J, Busuioc OA, Bero L: Industry sponsorship and research outcome. The Cochrane database of systematic reviews 2012; 12:MR000033.

9. Lund EM, James KM, Neaton JD: Veterinary randomized clinical trial reporting: a review of the small animal literature. J Vet Intern Med 1998; 12(2):57-60.

10. Sargeant JM, Thompson A, Valcour J, Elgie R, Saint-Onge J, Marcynuk P, Snedeker K: Quality of reporting of clinical trials of dogs and cats and associations with treatment effects. J Vet Intern Med 2010; 24(1):44-50.

11. O'Connor AM, Wellman NG, Rice M, Funk L: Characteristics of clinical trials assessing antimicrobial treatment of bovine respiratory disease, 1970-2005. Journal of the American Veterinary Medical Association 2010; 237(6):701-705.

12. Als-Nielsen B, Chen W, Gluud C, Kjaergard LL: Association of funding and conclusions in randomized drug trials: a reflection of treatment effect or adverse events? JAMA 2003; 290(7):921-928.

13. Sargeant JM, Elgie R, Valcour J, Saint-Onge J, Thompson A, Marcynuk P, Snedeker K: Methodological quality and completeness of reporting in clinical trials conducted in livestock species. Prev Vet Med 2009; 91(2-4):107-115.

14. Chan AW, Altman DG: Identifying outcome reporting bias in randomised trials on PubMed: review of publications and survey of authors. BMJ (Clinical research ed) 2005; 330(7494):753.

15. Dwan K, Altman DG, Arnaiz JA, Bloom J, Chan AW, Cronin E, Decullier E, Easterbrook PJ, Von Elm E, Gamble C, Ghersi D, Ioannidis JP, Simes J, Williamson PR: Systematic review of the empirical evidence of study publication bias and outcome reporting bias. PloS one 2008; 3(8):e3081.

16. Meerpohl JJ, Schell LK, Bassler D, Gallus S, Kleijnen J, Kulig M, La Vecchia C, Marušić A, Ravaud P, Reis A, Schmucker C, Strech D, Urrútia G, Wager E, Antes G: Evidence-informed recommendations to reduce dissemination bias in clinical research: conclusions from the OPEN (Overcome failure to Publish nEgative fiNdings) project based on an international consensus meeting. BMJ Open 2015; 5(5).

17. [www.alltrials.net](http://www.alltrials.net)

18. Dean R, Royle N, Boulton C, Turner S, McKenzie B, O'Connor A, Budsberg S, Pion P, Lambert A, Jarvis S, Reynolds S: Veterinary All Trials Initiative. Vet Rec 2015; 177(5):131-132.

19. Devereaux PJ, Choi PT, El-Dika S, Bhandari M, Montori VM, Schunemann HJ, Garg AX, Busse JW, Heels-Ansdell D, Ghali WA, Manns BJ, Guyatt GH: An observational study found that authors of randomized controlled trials frequently use concealment of randomization and blinding, despite the failure to report these methods. Journal of clinical epidemiology 2004; 57(12):1232-1236.

20. Hill CL, LaValley MP, T Felson D: Discrepancy between published report and actual conduct of randomized clinical trials. Journal of Clinical Epidemiology 2002; 55(8):783-786.

21. Moher D, Pham B, Jones A, Cook DJ, Jadad AR, Moher M, Tugwell P, Klassen TP: Does quality of reports of randomised trials affect estimates of intervention efficacy reported in meta-analyses? Lancet 1998; 352(9128):609-613.

22. Kjaergard LL, Villumsen J, Gluud C: Reported methodologic quality and discrepancies between large and small randomized trials in meta-analyses. Annals of internal medicine 2001; 135(11):982-989.

23. Begg C, Cho M, Eastwood S, Horton R, Moher D, Olkin I, Pitkin R, Rennie D, Schulz KF, Simel D, Stroup DF: Improving the quality of reporting of randomized controlled trials. The CONSORT statement. JAMA 1996; 276(8):637-639.

24. Simera I, Moher D, Hirst A, Hoey J, Schulz KF, Altman DG: Transparent and accurate reporting increases reliability, utility, and impact of your research: reporting guidelines and the EQUATOR Network. BMC medicine 2010; 8:24.

25. Turner L, Shamseer L, Altman DG, Schulz KF, Moher D: Does use of the CONSORT Statement impact the completeness of reporting of randomised controlled trials published in medical journals? A Cochrane review. Systematic reviews 2012; 1:60.

26. Plint AC, Moher D, Morrison A, Schulz K, Altman DG, Hill C, Gaboury I: Does the CONSORT checklist improve the quality of reports of randomised controlled trials? A systematic review. The Medical journal of Australia 2006; 185(5):263-267.

27. O'Connor AM, Sargeant JM, Gardner IA, Dickson JS, Torrence ME, Dewey CE, Dohoo IR, Evans RB, Gray JT, Greiner M, Keefe G, Lefebvre SL, Morley PS, Ramirez A, Sischo W, Smith DR, Snedeker K, Sofos J, Ward MP, Wills R: The REFLECT statement: methods and processes of creating reporting guidelines for randomized controlled trials for livestock and food safety. Prev Vet Med 2010; 93(1):11-18.

28. Sargeant JM, O'Connor AM, Gardner IA, Dickson JS, Torrence ME, Dohoo IR, Lefebvre SL, Morley PS, Ramirez A, Snedeker K: The REFLECT statement: reporting guidelines for randomized controlled trials in livestock and food safety: explanation and elaboration. Journal of food protection 2010; 73(3):579-603.

29. Schulz KF, Altman DG, Moher D: CONSORT 2010 statement: updated guidelines for reporting parallel group randomised trials. BMJ 2010; 340:c332.

30. Grindlay DJ, Dean RS, Christopher MM, Brennan ML: A survey of the awareness, knowledge, policies and views of veterinary journal Editors-in-Chief on reporting guidelines for publication of research. BMC Vet Res 2014; 10:10.

**Figure legends**

**Figure 1.** Summary of the number of papers retrieved from literature searches, numbers of papers excluded using Level 1 and 2 exclusion criteria and number of papers and individual trials analysed for each species and overall.

**Figure 2.** Percentages of all trials (N=126) at high, low or unclear risk of bias for the five criteria assessed.

**Table 1.** Two levels of inclusion and exclusion criteria applied to the search results

| **Level 1: Inclusion criteria for publications** | **Level 1: Exclusion criteria for publications** |
| --- | --- |
| Species of interest is cats, dogs, horses, cattle or sheep | Not about cats, dogs, horses, cattle or sheep |
| Published in 2011 | E published only in 2011 if full publication occurred in a different calendar year |
| RCT according to PubMed publication types and the Cochrane definition | Not an RCT (not indexed as an RCT by PubMed or not fulfilling Cochrane definition of an RCT) |
| Treatment of interest is a pharmaceutical intervention (including anthelmintics and vaccines) | Treatment of interest is not a pharmaceutical agent e.g. nutritional, surgical, animal husbandry etc |
| **Level 2: Inclusion criteria for analysis of pharmaceutical RCTs** | **Level 2: Exclusion criteria for analysis of pharmaceutical RCTs** |
| Primary aim is to assess efficacy | Primary aim was not to assess efficacy (pharmacokinetic/dynamic studies, safety studies, physiological effects, resistance testing, testing routes of administration only, testing timing of administration only) |
| Identifiable treatment or protocol of interest | Treatment or protocol of interest could not be identified |
| Single dose of the treatment of interest used | Multiple doses of the treatment of interest used/dose finding studies |
| Published in English | Not available in English |

**Table 2.** Number and funding source of papers and individual trials following level 2 exclusion criteria application

|  | **Number of cat papers (trials)** | **Number of dog papers (trials)** | **Number of horse papers (trials)** | **Number of cattle papers (trials)** | **Number of sheep papers (trials)** | **Total number of papers (trials, % of total trials)** |
| --- | --- | --- | --- | --- | --- | --- |
| Papers including pharmaceutical agent RCTs | 17 | 49 | 28 | 61 | 17 | **172** |
| Papers excluded from analysis* | 9 | 21 | 17 | 29 | 10 | **86** |
| Papers analysed | 8 (9 trials) | 28 (44 trials) | 11 (11 trials) | 32 (36 trials) | 7 (26 trials) | **86 (126 trials)** |
| **Funding sources of analysed papers** | | | | | | |
| Pharmaceutical company funded/pharmaceutical company involvement | 4 (5 trials) | 17 (33 trials) | 4 (4 trials) | 20 (23 trials) | 2 (21 trials) | **47 (86 trials; 68.3%)** |
| Non pharmaceutical funding stated | 3 (3 trials) | 4 (4 trials) | 2 (2 trials) | 6 ( 7 trials) | 3 (3 trials) | **18 (19 trials; 15.1%)** |
| No funding stated | 1 (1 trial) | 7 (7 trials) | 5 (5 trials) | 6 (6 trials) | 2 (2 trials) | **21 (21 trials; 16.7%)** |

Included studies are the pharmaceutical agent RCTs. * see Additional Table 1 for reasons for exclusions from analysis. There was no statistical difference (p=0.53) between funding sources between companion animal species (cats, dogs and horses) and farm animal species (cows and sheep)

**Table 3.** Categorisation of individual outcomes from 126 trials (960 outcomes)

|  | Outcomes from trials with pharmaceutical funding/involvement | Outcomes from trials with non-pharmaceutical funding stated | Outcomes from trials with no funding source stated | **Outcomes from all trials** |
| --- | --- | --- | --- | --- |
| Positive outcomes | 56.9% (339/596)_a_ | 34.9% (66/189)_b_ | 29.1% (51/175)_b_ | **47.5% (456/960)** |
| Negative outcomes | 23.5% (140/596)_a_ | 37.6% (71/189)_b_ | 37.1% (65/175)_b_ | **28.8% (276/960)** |
| No difference | 0.8% (5/596)_a_ | 2.6% (5/189)_a,b_ | 4.6% (8/175)_b_ | **1.9% (18/960)** |
| Described only | 12.8% (76/596) | 16.9% (32/189) | 18.9% (33/175) | **14.7% (141/960)** |
| Not reported | 6.0% (36/596) | 7.9% (15/189) | 10.3% (18/175) | **7.2% (69/960)** |

Data shown as percentages and raw numbers in brackets. Significant differences (p<0.05) existing between funding categories within rows are indicated by differing subscript letters. (No subscript letters in a row signifiy no significant differences. The presence of a subscript letter (e.g. ‘a’0 in a cell indicates that it is significantly different from a cell marked with a different letter (e.g. ‘b’). If a cell has two subscript letters (e.g. ‘a,b’) then it is different from cells individually marked with each letter.)

**Table 4.** Risk of bias for trials within different funding categories and overall

|  | **Risk of bias** | **Pharmaceutical funding/involvement (86 trials)** | **Non pharmaceutical funding declared (19 trials)** | **No funding source declared (21 trials)** | **All trials (126 trials)** |
| --- | --- | --- | --- | --- | --- |
| **Sequence generation** | High | 3 (3.5%) | 0 (0%) | 0 (0%) | **3 (2.4%)** |
|  | Low | 16 (18.6%) | 7 (36.8%) | 8 (38.1%) | **31 (24.6%)** |
|  | Unclear | 67 (77.9%) | 12 (63.2%) | 13 (61.9%) | **92 (73.0%)** |
| **Allocation concealment** | High | 5 (5.8%) | 1 (5.3%) | 0 (0%) | **6 (4.8%)** |
|  | Low | 5 (5.8%) | 3 (15.8%) | 3 (14.3%) | **11 (8.7%)** |
|  | Unclear | 76 (88.4%) | 15 (78.9%) | 18 (85.7%) | **109 (86.5%)** |
| **Blinding** | High | 5 (5.8%) | 3 (15.8%) | 2 (9.5%) | **10 (7.9%)** |
|  | Low | 29 (33.7%) | 7 (36.8%) | 8 (38.1%) | **44 (34.9%)** |
|  | Unclear | 52 (60.5%) | 9 (47.4%) | 11 (52.4%) | **72 (57.1%)** |
| **Incomplete outcome reporting** | High | 14 (16.3%) | 1 (5.3%) | 4 (19.0%) | **19 (15.1%)** |
|  | Low | 36 (41.9%) | 15 (78.9%) | 14 (66.7%) | **65 (51.6%)** |
|  | Unclear | 36 (41.9%) | 3 (15.8%) | 3 (14.3%) | **42 (33.3%)** |
| **Selective outcome reporting** | High | 18 (20.9%) | 5 (26.3%) | 6 (28.6%) | **29 (23.0%)** |
|  | Low | 64 (74.4%) | 11 (57.9%) | 12 (57.1%) | **87 (69.0%)** |
|  | Unclear | 4 (4.7%) | 3 (15.8%) | 3 (14.3%) | **10 (7.9%)** |

Data expressed as as raw numbers and percentages of total trials.

**Additional files (all below)**

File name: Additional table 1

File title:–Reasons for exclusions of RCTs involving pharmaceutical agents from analysis

Type of data: Table containing numbers of trials excluded for each reason organised in species groups

File name: Additional file 1 –

File title:Cochrane (<http://www.cochrane.org/glossary/>) definitions of types of bias

Type of data: Written descriptions of the definitions of the Cochrane types of bias

File name:Additional file 2.

File title: References for all papers included in the analysis within this study (single dose efficacy studies of pharmaceutical interventions in cats, dogs, horses, cattle or sheep published in 2011)

Type of data: List of references in word

1. Abelson AL, Armitage-Chan E, Lindsey JC, Wetmore LA: A comparison of epidural morphine with low dose bupivacaine versus epidural morphine alone on motor and respiratory function in dogs following splenectomy. Veterinary anaesthesia and analgesia 2011, 38(3):213-223.

2. Agaoglu AR, Schafer-Somi S, Kaya D, Kucukaslan I, Emre B, Gultiken N, Mulazimoglu BS, Colak A, Aslan S: The intravaginal application of misoprostol improves induction of abortion with aglepristone. Theriogenology 2011, 76(1):74-82.

3. Aguado D, Benito J, Gomez de Segura IA: Reduction of the minimum alveolar concentration of isoflurane in dogs using a constant rate of infusion of lidocaine-ketamine in combination with either morphine or fentanyl. Veterinary journal (London, England : 1997) 2011, 189(1):63-66.

4. Allen KJ, Rogan D, Finlay BB, Potter AA, Asper DJ: Vaccination with type III secreted proteins leads to decreased shedding in calves after experimental infection with Escherichia coli O157. Canadian journal of veterinary research = Revue canadienne de recherche veterinaire 2011, 75(2):98-105.

5. Altreuther G, Gasda N, Adler K, Hellmann K, Thurieau H, Schimmel A, Hutchens D, Krieger KJ: Field evaluations of the efficacy and safety of Emodepside plus toltrazuril (Procox(R) oral suspension for dogs) against naturally acquired nematode and Isospora spp. infections in dogs. Parasitology research 2011, 109 Suppl 1:S21-28.

6. Altreuther G, Gasda N, Schroeder I, Joachim A, Settje T, Schimmel A, Hutchens D, Krieger KJ: Efficacy of emodepside plus toltrazuril suspension (Procox((R)) oral suspension for dogs) against prepatent and patent infection with Isospora canis and Isospora ohioensis-complex in dogs. Parasitology research 2011, 109 Suppl 1:S9-20.

7. Avendano-Reyes L, Macias-Cruz U, Alvarez-Valenzuela FD, Aguila-Tepato E, Torrentera-Olivera NG, Soto-Navarro SA: Effects of zilpaterol hydrochloride on growth performance, carcass characteristics, and wholesale cut yield of hair-breed ewe lambs consuming feedlot diets under moderate environmental conditions. Journal of animal science 2011, 89(12):4188-4194.

8. Baggott D, Casartelli A, Fraisse F, Manavella C, Marteau R, Rehbein S, Wiedemann M, Yoon S: Demonstration of the metaphylactic use of gamithromycin against bacterial pathogens associated with bovine respiratory disease in a multicentre farm trial. The Veterinary record 2011, 168(9):241.

9. Bergamasco L, Coetzee JF, Gehring R, Murray L, Song T, Mosher RA: Effect of intravenous sodium salicylate administration prior to castration on plasma cortisol and electroencephalography parameters in calves. Journal of veterinary pharmacology and therapeutics 2011, 34(6):565-576.

10. Bettschart-Wolfensberger R, Dicht S, Vullo C, Frotzler A, Kuemmerle JM, Ringer SK: A clinical study on the effect in horses during medetomidine-isoflurane anaesthesia, of butorphanol constant rate infusion on isoflurane requirements, on cardiopulmonary function and on recovery characteristics. Veterinary anaesthesia and analgesia 2011, 38(3):186-194.

11. Beugnet F, Doyle V, Murray M, Chalvet-Monfray K: Comparative efficacy on dogs of a single topical treatment with the pioneer fipronil/(S)-methoprene and an oral treatment with spinosad against Ctenocephalides felis. Parasite (Paris, France) 2011, 18(4):325-331.

12. Bryan MA, Heuer C, Emslie FR: The comparative efficacy of two long-acting dry-cow cephalonium products in curing and preventing intramammary infections. New Zealand veterinary journal 2011, 59(4):166-173.

13. Buckley GJ, Rozanski EA, Rush JE: Randomized, blinded comparison of epinephrine and vasopressin for treatment of naturally occurring cardiopulmonary arrest in dogs. Journal of veterinary internal medicine / American College of Veterinary Internal Medicine 2011, 25(6):1334-1340.

14. Cadot P, Hensel P, Bensignor E, Hadjaje C, Marignac G, Beco L, Fontaine J, Jamet JF, Georgescu G, Campbell K et al: Masitinib decreases signs of canine atopic dermatitis: a multicentre, randomized, double-blind, placebo-controlled phase 3 trial. Veterinary dermatology 2011, 22(6):554-564.

15. Camargo JB, Steagall PV, Minto BW, Lorena SE, Mori ES, Luna SP: Post-operative analgesic effects of butorphanol or firocoxib administered to dogs undergoing elective ovariohysterectomy. Veterinary anaesthesia and analgesia 2011, 38(3):252-259.

16. Cohn LA, Birkenheuer AJ, Brunker JD, Ratcliff ER, Craig AW: Efficacy of atovaquone and azithromycin or imidocarb dipropionate in cats with acute cytauxzoonosis. Journal of veterinary internal medicine / American College of Veterinary Internal Medicine 2011, 25(1):55-60.

17. Congdon JM, Marquez M, Niyom S, Boscan P: Evaluation of the sedative and cardiovascular effects of intramuscular administration of dexmedetomidine with and without concurrent atropine administration in dogs. Journal of the American Veterinary Medical Association 2011, 239(1):81-89.

18. Davey RB, Pound JM, Klavons JA, Lohmeyer KH, Freeman JM, Perez de Leon AA, Miller RJ: Efficacy and blood sera analysis of a long-acting formulation of moxidectin against Rhipicephalus (Boophilus) microplus (Acari: Ixodidae) on treated cattle. Journal of medical entomology 2011, 48(2):314-321.

19. Dodd CC, Renter DG, Thomson DU, Nagaraja TG: Evaluation of the effects of a commercially available Salmonella Newport siderophore receptor and porin protein vaccine on fecal shedding of Salmonella bacteria and health and performance of feedlot cattle. American journal of veterinary research 2011, 72(2):239-247.

20. Faya M, Carranza A, Priotto M, Graiff D, Zurbriggen G, Diaz JD, Gobello C: Long-term melatonin treatment prolongs interestrus, but does not delay puberty, in domestic cats. Theriogenology 2011, 75(9):1750-1754.

21. Felix TL, Loerch SC: Effects of haylage and monensin supplementation on performance, carcass characteristics, and ruminal metabolism of feedlot cattle fed diets containing 60% dried distillers grains. Journal of animal science 2011, 89(8):2614-2623.

22. Fischer Y, Ritz S, Weber K, Sauter-Louis C, Hartmann K: Randomized, placebo controlled study of the effect of propentofylline on survival time and quality of life of cats with feline infectious peritonitis. Journal of veterinary internal medicine / American College of Veterinary Internal Medicine 2011, 25(6):1270-1276.

23. Fourie JJ, Beugnet F, Ollagnier C, Pollmeier MG: Study of the sustained speed of kill of the combination of fipronil/amitraz/(S)-methoprene and the combination of imidacloprid/permethrin against Dermacentor reticulatus, the European dog tick. Parasite (Paris, France) 2011, 18(4):319-323.

24. Friedman E, Voet H, Reznikov D, Dagoni I, Roth Z: Induction of successive follicular waves by gonadotropin-releasing hormone and prostaglandin F(2alpha) to improve fertility of high-producing cows during the summer and autumn. Journal of dairy science 2011, 94(5):2393-2402.

25. Gabriel HG, Wallenhorst S, Dietrich E, Holtz W: The effect of prostaglandin F(2alpha) administration at the time of insemination on the pregnancy rate of dairy cows. Animal reproduction science 2011, 123(1-2):1-4.

26. Geary TW, Wells KJ, deAvila DM, deAvila J, Conforti VA, McLean DJ, Roberts AJ, Waterman RW, Reeves JJ: Effects of immunization against luteinizing hormone-releasing hormone and treatment with trenbolone acetate on reproductive function of beef bulls and steers. Journal of animal science 2011, 89(7):2086-2095.

27. Gordon-Evans WJ, Dunning D, Johnson AL, Knap KE: Effect of the use of carprofen in dogs undergoing intense rehabilitation after lateral fabellar suture stabilization. Journal of the American Veterinary Medical Association 2011, 239(1):75-80.

28. Gruet P, Seewald W, King JN: Evaluation of subcutaneous and oral administration of robenacoxib and meloxicam for the treatment of acute pain and inflammation associated with orthopedic surgery in dogs. American journal of veterinary research 2011, 72(2):184-193.

29. Habing GG, Neuder LM, Raphael W, Piper-Youngs H, Kaneene JB: Efficacy of oral administration of a modified-live Salmonella Dublin vaccine in calves. Journal of the American Veterinary Medical Association 2011, 238(9):1184-1190.

30. Hardie EM, Lascelles BD, Meuten T, Davidson GS, Papich MG, Hansen BD: Evaluation of intermittent infusion of bupivacaine into surgical wounds of dogs postoperatively. Veterinary journal (London, England : 1997) 2011, 190(2):287-289.

31. Hellmann K, Heine J, Braun G, Paran-Dobesova R, Svobodova V: Evaluation of the therapeutic and preventive efficacy of 2.5 % moxidectin / 10 % imidacloprid (Advocate((R)), Bayer animal health) in dogs naturally infected or at risk of natural infection by Dirofilaria repens. Parasitology research 2011, 109 Suppl 1:S77-86.

32. Hennet PR, Camy GA, McGahie DM, Albouy MV: Comparative efficacy of a recombinant feline interferon omega in refractory cases of calicivirus-positive cats with caudal stomatitis: a randomised, multi-centre, controlled, double-blind study in 39 cats. Journal of feline medicine and surgery 2011, 13(8):577-587.

33. Hermo GA, Turic E, Angelico D, Scursoni AM, Gomez DE, Gobello C, Alonso DF: Effect of adjuvant perioperative desmopressin in locally advanced canine mammary carcinoma and its relation to histologic grade. Journal of the American Animal Hospital Association 2011, 47(1):21-27.

34. Heuwieser W, Iwersen M, Goetze L: Efficacy of carprofen on conception rates in lactating dairy cows after subcutaneous or intrauterine administration at the time of breeding. Journal of dairy science 2011, 94(1):146-151.

35. Horohov DW, Loynachan AT, Page AE, Hughes K, Timoney JF, Fettinger M, Hatch T, Spaulding JG, McMichael J: The use of streptolysin O (SLO) as an adjunct therapy for Rhodococcus equi pneumonia in foals. Veterinary microbiology 2011, 154(1-2):156-162.

36. Johnston TP, Mondal P, Pal D, MacGee S, Stromberg AJ, Alur H: Canine periodontal disease control using a clindamycin hydrochloride gel. Journal of veterinary dentistry 2011, 28(4):224-229.

37. Jonsson NN, Piper EK, Gray CP, Deniz A, Constantinoiu CC: Efficacy of toltrazuril 5 % suspension against Eimeria bovis and Eimeria zuernii in calves and observations on the associated immunopathology. Parasitology research 2011, 109 Suppl 1:S113-128.

38. Kasravi R, Bolourchi M, Farzaneh N, Seifi HA, Barin A, Hovareshti P, Gharagozlou F: Efficacy of conventional and extended intra-mammary treatment of persistent sub-clinical mastitis with cefquinome in lactating dairy cows. Tropical animal health and production 2011, 43(6):1203-1210.

39. Kilpinen S, Spillmann T, Syrja P, Skrzypczak T, Louhelainen M, Westermarck E: Effect of tylosin on dogs with suspected tylosin-responsive diarrhea: a placebo-controlled, randomized, double-blinded, prospective clinical trial. Acta veterinaria Scandinavica 2011, 53:26.

40. Kloppel H, Leece EA: Comparison of ketamine and alfaxalone for induction and maintenance of anaesthesia in ponies undergoing castration. Veterinary anaesthesia and analgesia 2011, 38(1):37-43.

41. Knights M, Ramgattie R, Siew N, Singh-Knights D, Bourne G: Effectiveness of a short-term treatment with progesterone injections on synchrony of lambing and fertility in tropical hair sheep. Animal reproduction science 2011, 126(1-2):70-75.

42. Lawrence TE, Gasch CA, Hutcheson JP, Hodgen JM: Zilpaterol improves feeding performance and fabrication yield of concentrate-finished cull cows. Journal of animal science 2011, 89(7):2170-2175.

43. Levy JK, Friary JA, Miller LA, Tucker SJ, Fagerstone KA: Long-term fertility control in female cats with GonaCon, a GnRH immunocontraceptive. Theriogenology 2011, 76(8):1517-1525.

44. Little PR, Hodge A, Maeder SJ, Wirtherle NC, Nicholas DR, Cox GG, Conder GA: Efficacy of a combined oral formulation of derquantel-abamectin against the adult and larval stages of nematodes in sheep, including anthelmintic-resistant strains. Veterinary parasitology 2011, 181(2-4):180-193.

45. Ma J, Shi N, Jiang CG, Lin YZ, Wang XF, Wang S, Lv XL, Zhao LP, Shao YM, Kong XG et al: A proviral derivative from a reference attenuated EIAV vaccine strain failed to elicit protective immunity. Virology 2011, 410(1):96-106.

46. Macrina AL, Tozer PR, Kensinger RS: Induced lactation in pubertal heifers: efficacy, response to bovine somatotropin, and profitability. Journal of dairy science 2011, 94(3):1355-1364.

47. Marino CT, Otero WG, Rodrigues PH, Dicostanzo A, Millen DD, Pacheco RL, Dilorenzo N, Martins CL, Arrigoni MD: Effects of adding polyclonal antibody preparations on ruminal fermentation patterns and digestibility of cows fed different energy sources. Journal of animal science 2011, 89(10):3228-3235.

48. Marquezini GH, Dahlen CR, Bird SL, Lamb GC: Administration of human chorionic gonadotropin to suckled beef cows before ovulation synchronization and fixed-time insemination: replacement of gonadotropin-releasing hormone with human chorionic gonadotropin. Journal of animal science 2011, 89(10):3030-3039.

49. Martins JP, Policelli RK, Neuder LM, Raphael W, Pursley JR: Effects of cloprostenol sodium at final prostaglandin F2alpha of Ovsynch on complete luteolysis and pregnancy per artificial insemination in lactating dairy cows. Journal of dairy science 2011, 94(6):2815-2824.

50. McArt JA, Nydam DV, Ospina PA, Oetzel GR: A field trial on the effect of propylene glycol on milk yield and resolution of ketosis in fresh cows diagnosed with subclinical ketosis. Journal of dairy science 2011, 94(12):6011-6020.

51. McClure S, Sibert G, Hallberg J, Bade D: Efficacy of a 2-dose regimen of a sustained release ceftiofur suspension in horses with Streptococcus equi subsp. zooepidemicus bronchopneumonia. Journal of veterinary pharmacology and therapeutics 2011, 34(5):442-447.

52. Mellett AM, Nakamura RK, Bianco D: A prospective study of clopidogrel therapy in dogs with primary immune-mediated hemolytic anemia. Journal of veterinary internal medicine / American College of Veterinary Internal Medicine 2011, 25(1):71-75.

53. Merino O, Almazan C, Canales M, Villar M, Moreno-Cid JA, Estrada-Pena A, Kocan KM, de la Fuente J: Control of Rhipicephalus (Boophilus) microplus infestations by the combination of subolesin vaccination and tick autocidal control after subolesin gene knockdown in ticks fed on cattle. Vaccine 2011, 29(12):2248-2254.

54. Meyers-Brown G, Bidstrup LA, Famula TR, Colgin M, Roser JF: Treatment with recombinant equine follicle stimulating hormone (reFSH) followed by recombinant equine luteinizing hormone (reLH) increases embryo recovery in superovulated mares. Animal reproduction science 2011, 128(1-4):52-59.

55. Morton CM, Grant D, Johnston L, Letellier IM, Narbe R: Clinical evaluation of meloxicam versus ketoprofen in cats suffering from painful acute locomotor disorders. Journal of feline medicine and surgery 2011, 13(4):237-243.

56. O'Connor AM, Brace S, Gould S, Dewell R, Engelken T: A randomized clinical trial evaluating a farm-of-origin autogenous Moraxella bovis vaccine to control infectious bovine keratoconjunctivis (pinkeye) in beef cattle. Journal of veterinary internal medicine / American College of Veterinary Internal Medicine 2011, 25(6):1447-1453.

57. Olivera-Muzante J, Fierro S, Lopez V, Gil J: Comparison of prostaglandin- and progesterone-based protocols for timed artificial insemination in sheep. Theriogenology 2011, 75(7):1232-1238.

58. Olsen L, Bondesson U, Brostrom H, Olsson U, Mazogi B, Sundqvist M, Tjalve H, Ingvast-Larsson C: Pharmacokinetics and effects of cetirizine in horses with insect bite hypersensitivity. Veterinary journal (London, England : 1997) 2011, 187(3):347-351.

59. Pang WY, Earley B, Murray M, Sweeney T, Gath V, Crowe MA: Banding or Burdizzo castration and carprofen administration on peripheral leukocyte inflammatory cytokine transcripts. Research in veterinary science 2011, 90(1):127-132.

60. Parr SL, Chung KY, Hutcheson JP, Nichols WT, Yates DA, Streeter MN, Swingle RS, Galyean ML, Johnson BJ: Dose and release pattern of anabolic implants affects growth of finishing beef steers across days on feed. Journal of animal science 2011, 89(3):863-873.

61. Pasa S, Voyvoda H, Karagenc T, Atasoy A, Gazyagci S: Failure of combination therapy with imidocarb dipropionate and toltrazuril to clear Hepatozoon canis infection in dogs. Parasitology research 2011, 109(3):919-926.

62. Pinard CL, Gauvin D, Moreau M, Martel-Pelletier J, Pelletier JP, Troncy E: Measurements of canine aqueous humor inflammatory mediators and the effect of carprofen following anterior chamber paracentesis. Veterinary ophthalmology 2011, 14(5):296-303.

63. Psatha E, Alibhai HI, Jimenez-Lozano A, Armitage-Chan E, Brodbelt DC: Clinical efficacy and cardiorespiratory effects of alfaxalone, or diazepam/fentanyl for induction of anaesthesia in dogs that are a poor anaesthetic risk. Veterinary anaesthesia and analgesia 2011, 38(1):24-36.

64. Redmond JS, Macedo GG, Velez IC, Caraty A, Williams GL, Amstalden M: Kisspeptin activates the hypothalamic-adenohypophyseal-gonadal axis in prepubertal ewe lambs. Reproduction (Cambridge, England) 2011, 141(4):541-548.

65. Reist M, Forbes AB, Bonfanti M, Beretta W, Pfister K: Effect of eprinomectin treatment on milk yield and quality in dairy cows in South Tyrol, Italy. The Veterinary record 2011, 168(18):484.

66. Santos LC, Ludders JW, Erb HN, Martin-Flores M, Basher KL, Kirch P: A randomized, blinded, controlled trial of the antiemetic effect of ondansetron on dexmedetomidine-induced emesis in cats. Veterinary anaesthesia and analgesia 2011, 38(4):320-327.

67. Sawalha MN, Kridli RT, Jawasreh KI, Meza-Herrera CA: The use of melatonin and progestagen-eCG to initiate reproductive activity in prepuberal Awassi ewe lambs. Tropical animal health and production 2011, 43(7):1345-1350.

68. Schauer SN, Briant C, Ottogalli M, Decourt C, Handel IG, Donadeu FX: Supplementation of equine early spring transitional follicles with luteinizing hormone stimulates follicle growth but does not restore steroidogenic activity. Theriogenology 2011, 75(6):1076-1084.

69. Schauvliege S, Marcilla MG, Verryken K, Duchateau L, Devisscher L, Gasthuys F: Effects of a constant rate infusion of detomidine on cardiovascular function, isoflurane requirements and recovery quality in horses. Veterinary anaesthesia and analgesia 2011, 38(6):544-554.

70. Schimmel A, Schroeder I, Altreuther G, Settje T, Charles S, Wolken S, Kok DJ, Ketzis J, Young D, Hutchens D et al: Efficacy of emodepside plus toltrazuril (Procox((R)) oral suspension for dogs) against Toxocara canis, Uncinaria stenocephala and Ancylostoma caninum in dogs. Parasitology research 2011, 109 Suppl 1:S1-8.

71. Schukken YH, Bennett GJ, Zurakowski MJ, Sharkey HL, Rauch BJ, Thomas MJ, Ceglowski B, Saltman RL, Belomestnykh N, Zadoks RN: Randomized clinical trial to evaluate the efficacy of a 5-day ceftiofur hydrochloride intramammary treatment on nonsevere gram-negative clinical mastitis. Journal of dairy science 2011, 94(12):6203-6215.

72. Schuller S, Van Israel N, Vanbelle S, Clercx C, McEntee K: Lack of efficacy of low-dose spironolactone as adjunct treatment to conventional congestive heart failure treatment in dogs. Journal of veterinary pharmacology and therapeutics 2011, 34(4):322-331.

73. Siedek EM, Schmidt H, Sture GH, Raue R: Vaccination with canine parvovirus type 2 (CPV-2) protects against challenge with virulent CPV-2b and CPV-2c. Berliner und Munchener tierarztliche Wochenschrift 2011, 124(1-2):58-64.

74. Snyder DE, Wiseman S, Bowman DD, McCall JW, Reinemeyer CR: Assessment of the effectiveness of a combination product of spinosad and milbemycin oxime on the prophylaxis of canine heartworm infection. Veterinary parasitology 2011, 180(3-4):262-266.

75. Sturgill TL, Giguere S, Franklin RP, Cohen ND, Hagen J, Kalyuzhny AE: Effects of inactivated parapoxvirus ovis on the cumulative incidence of pneumonia and cytokine secretion in foals on a farm with endemic infections caused by Rhodococcus equi. Veterinary immunology and immunopathology 2011, 140(3-4):237-243.

76. Teske E, Rutteman GR, Kirpenstein J, Hirschberger J: A randomized controlled study into the efficacy and toxicity of pegylated liposome encapsulated doxorubicin as an adjuvant therapy in dogs with splenic haemangiosarcoma. Veterinary and comparative oncology 2011, 9(4):283-289.

77. Thiry J, Rubion S, Sarasola P, Bonnier M, Hartmann M, de Haas V: Efficacy and safety of a new 450 mg/ml florfenicol formulation administered intramuscularly in the treatment of bacterial bovine respiratory disease. The Veterinary record 2011, 169(20):526.

78. Thomasy SM, Lim CC, Reilly CM, Kass PH, Lappin MR, Maggs DJ: Evaluation of orally administered famciclovir in cats experimentally infected with feline herpesvirus type-1. American journal of veterinary research 2011, 72(1):85-95.

79. Trotz-Williams LA, Jarvie BD, Peregrine AS, Duffield TF, Leslie KE: Efficacy of halofuginone lactate in the prevention of cryptosporidiosis in dairy calves. The Veterinary record 2011, 168(19):509.

80. Unterer S, Strohmeyer K, Kruse BD, Sauter-Louis C, Hartmann K: Treatment of aseptic dogs with hemorrhagic gastroenteritis with amoxicillin/clavulanic acid: a prospective blinded study. Journal of veterinary internal medicine / American College of Veterinary Internal Medicine 2011, 25(5):973-979.

81. VanLeeuwen JA, Greenwood S, Clark F, Acorn A, Markham F, McCarron J, O'Handley R: Monensin use against Neospora caninum challenge in dairy cattle. Veterinary parasitology 2011, 175(3-4):372-376.

82. Vasconcelos JL, Sa Filho OG, Justolin PL, Morelli P, Aragon FL, Veras MB, Soriano S: Effects of postbreeding gonadotropin treatments on conception rates of lactating dairy cows subjected to timed artificial insemination or embryo transfer in a tropical environment. Journal of dairy science 2011, 94(1):223-234.

83. Veronesi F, Diaferia M, Viola O, Fioretti DP: Long-term effect of toltrazuril on growth performances of dairy heifers and beef calves exposed to natural Eimeria zuernii and Eimeria bovis infections. Veterinary journal (London, England : 1997) 2011, 190(2):296-299.

84. Villalba M, Santiago I, Gomez de Segura IA: Effects of constant rate infusion of lidocaine and ketamine, with or without morphine, on isoflurane MAC in horses. Equine veterinary journal 2011, 43(6):721-726.

85. von Krueger X, Heuwieser W: Effect of CIDR(R) on 4-day-service-rate, pregnancy rate and vaginal irritation in dairy heifers. Tierarztliche Praxis Ausgabe G, Grosstiere/Nutztiere 2011, 39(5):277-280.

86. Wall R, Bates P: Sheep scab control using trans-cinnamic acid. Veterinary parasitology 2011, 175(1-2):129-134.
